# α,β-D-Constrained Nucleic Acids Are Strong Terminators of Thermostable DNA Polymerases in Polymerase Chain Reaction

**DOI:** 10.1371/journal.pone.0025510

**Published:** 2011-10-03

**Authors:** Olivier Martínez, Vincent Ecochard, Sabrina Mahéo, Grégori Gross, Pierre Bodin, Justin Teissié, Jean-Marc Escudier, Laurent Paquereau

**Affiliations:** 1 Institut de Pharmacologie et de Biologie Structurale, Unité Mixte de Recherche 5089, Centre National de la Recherche Scientifique, Toulouse, France; 2 Université Paul Sabatier Toulouse III, Faculté des Sciences et d'Ingénierie, Toulouse, France; 3 Laboratoire de Synthèse et Physico-Chimie de Molécules d'Intérêt Biologique, Unité Mixte de Recherche 5068, Centre National de la Recherche Scientifique, Toulouse , France; German Cancer Research Center, Germany

## Abstract

(*S*_C5′_, R*_P_*) α,β-D- Constrained Nucleic Acids (CNA) are dinucleotide building blocks that can feature either B-type torsional angle values or non-canonical values, depending on their 5′C and P absolute stereochemistry. These CNA are modified neither on the nucleobase nor on the sugar structure and therefore represent a new class of nucleotide with specific chemical and structural characteristics. They promote marked bending in a single stranded DNA so as to preorganize it into a loop-like structure, and they have been shown to induce rigidity within oligonucleotides. Following their synthesis, studies performed on CNA have only focused on the constraints that this family of nucleotides introduced into DNA. On the assumption that bending in a DNA template may produce a terminator structure, we investigated whether CNA could be used as a new strong terminator of polymerization in PCR. We therefore assessed the efficiency of CNA as a terminator in PCR, using triethylene glycol phosphate units as a control. Analyses were performed by denaturing gel electrophoresis and several PCR products were further analysed by sequencing. The results showed that the incorporation of only one CNA was always skipped by the polymerases tested. On the other hand, two CNA units always stopped proofreading polymerases, such as *Pfu* DNA polymerase, as expected for a strong replication terminator. Non-proofreading enzymes, *e.g. Taq* DNA polymerase, did not recognize this modification as a strong terminator although it was predominantly stopped by this structure. In conclusion, this first functional use of CNA units shows that these modified nucleotides can be used as novel polymerization terminators of proofreading polymerases. Furthermore, our results lead us to propose that CNA and their derivatives could be useful tools for investigating the behaviour of different classes of polymerases.

## Introduction

The ability of the different thermostable DNA polymerases to perform synthesis using either modified DNA templates or modified nucleotides as precursors is a subject of considerable interest. Indeed, there is a rich literature concerning the bypass and/or the incorporation of non-natural DNA nucleotides. These modified nucleotides have been used to analyse the active site of DNA polymerase by insertion kinetics experiments, to protect nucleic acids against nuclease digestion, to perform mutagenesis, or to generate terminator structures. The modifications studied concern the sugar moiety (*e.g.*, 1,5-Anhydrohexitol Nucleotides [1], Glycerol-Nucleoside Triphosphates (GNA) [2], [3], α-L-*Threo-*furanosyl Nucleic Acids (TNA) [4], [5], 4′-acetylated thymidine triphosphate [6], Cyclohexenyl Nucleic Acid (CeNA) [7], Locked Nucleic Acid (LNA) [8], [9], and 2′,4′-Bridged Nucleotide Acids (BNA) [10], [11]) or the base moiety (*e.g.*, non-polar nucleotide analogues [12], abasic sites [13], and cage nucleotides [14], [15]). Some of these modified nucleotides were described as terminators of replication and their ability to stop polymerisation differed depending on the DNA polymerase used. *In vitro*, particularly in PCR experiments, the most common and the strengthened terminator is a non-nucleotide terminator composed of two successive triethylene glycol phosphate units incorporated within one of the two primers in a central position [16]. This primer will therefore have a tripartite composition (5′ lengthener-terminator-complementary sequence). These non-nucleotide terminators were designed to generate two strands that significantly differ in length and which can therefore be readily purified by gel electrophoresis.

One of the research domains in which these terminators are of particular interest is the selection of DNA aptamers. This selection is performed by systematically evolution of ligands by exponential enrichment, which is termed the SELEX method [17], [18]. Several techniques focused on easily and efficiently purifying the sense single-stranded DNA for each run of selection. The most common methods used are asymmetric PCR [19], lambda exonuclease digestion [20], magnetic separation with streptavidin-coated beads [21], and denaturing polyacrylamide gel electrophoresis [16]. Each of these methods has their specific drawbacks, although all, with the exception of purification by gel electrophoresis, are unable to ensure that purification of only the sense single-strand DNA was performed. Modification of the length of one strand obtained in PCR remains the most suitable means to obtain the desired single strand, even if the efficiency of the extraction of the single strand from the gel remains a limitation of this approach.

**Figure 1:**
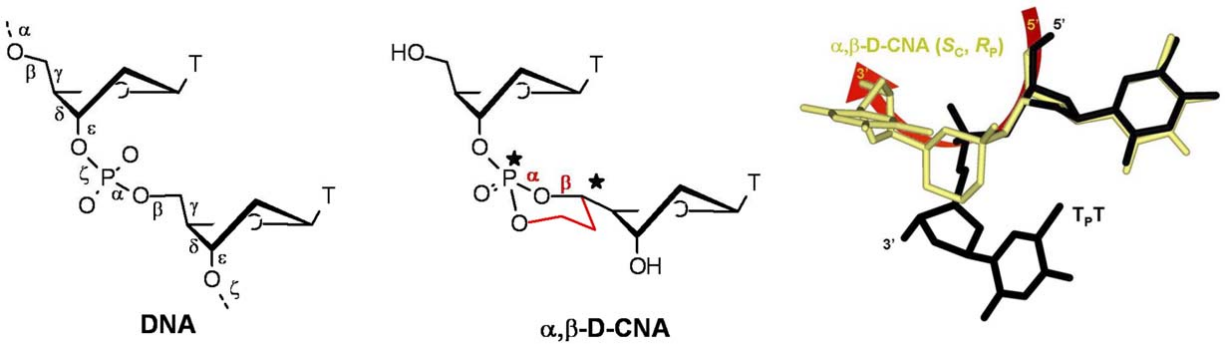
Structural constraints in (S_C5′_, R_P_) α,β-D-CNA TT. Left: the six backbone torsion angles (labelled α to ζ) of nucleic acids. Middle: the α,β-D-CNA TT dinucleotide in which α and β are stereocontrolled by a dioxaphosphorinane ring structure. Right: superimposition of the X-ray crystallography structure of unmodified TpT (black) [40] and NMR structure of (S_C5′_, R_P_) α,β-D-CNA TT (gold).

In the present study, we were focused on a class of recently described nucleotides, the (*S*_C5′_, R*_P_*) α,β-D-CNA TT (CNA). The nucleobase and the sugar structure are unmodified in these CNA, which represent a new class of nucleotide with specific chemical and structural characteristics. CNA are dinucleotide building blocks that can feature either B-type torsional angle values or non-canonical values, depending on their 5′C and P absolute stereochemistry (Fig. 1) [22], [23]. CNA display the *gauche* (+) conformation for the α torsional angle, which can induce destabilization of a duplex [24] as well as promote marked bending in a single-stranded DNA to preorganize it into a loop-like structure [25]. The conformational restriction of the phosphodiester linkage, by its transformation into a neutral stereocontrolled dioxaphosphorinane ring structure, stabilized DNA hairpin structures [26], [27] and induced rigidity within oligonucleotides [28].

We hypothesized that bending in the template may produce a terminator structure, and we tested whether CNA could be used as a new strong terminator of polymerization in PCR. We showed that a double CNA repeat completely stops thermostable DNA polymerases whereas one CNA is skipped. Our results also lead us to propose that CNA and their derivatives could be useful tools for investigating the behaviour of the different classes of polymerases.

## Results

### One α,β-D-CNA TT unit is skipped by DNA polymerases

**Figure 2:**
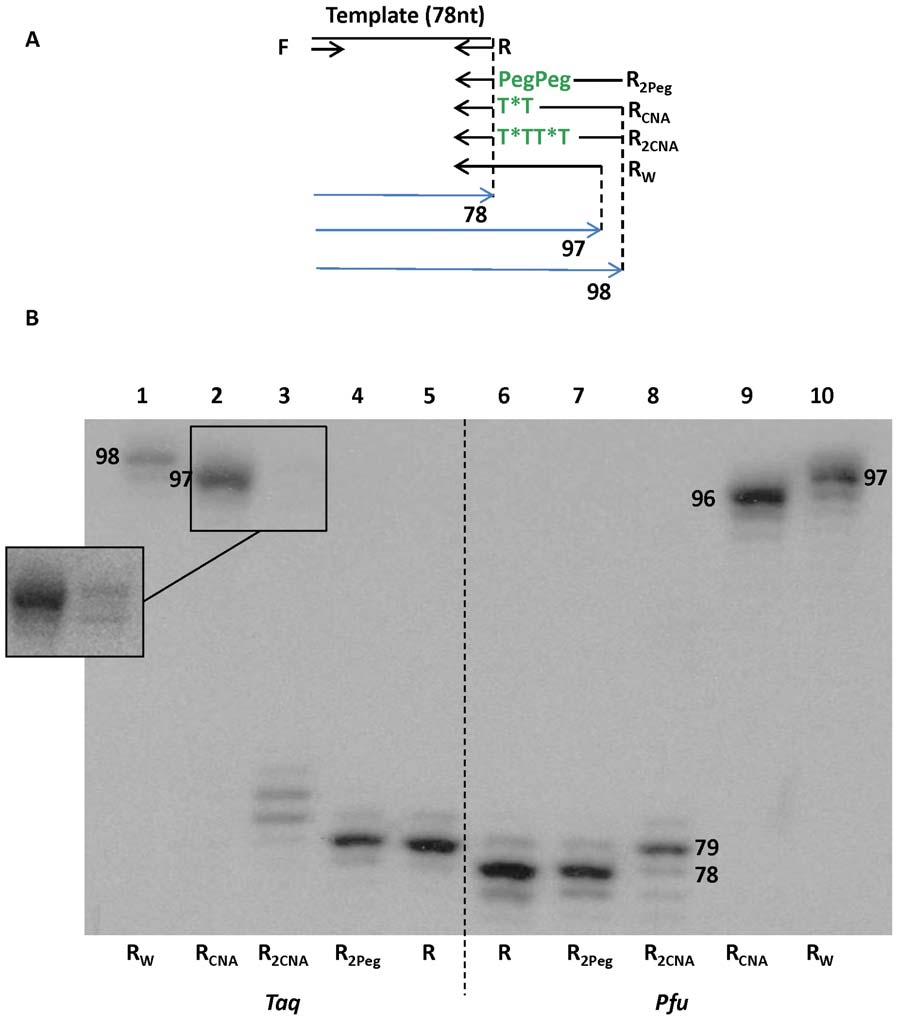
CNA as terminator of polymerization. A) Set of primers used for each PCR. Forward primer was ^32^P labelled to detect only the sense strand. The lengths of the PCR products expected are indicated. B) Denaturing polyacrylamide gel electrophoresis obtained for each set of primers with *Taq* DNA polymerase (1–5) and *Pfu* DNA polymerase (6–10). For each polymerase, the PCR were performed with different reverse primers: **R_W_** (lanes 1, 10); **R_CNA_** (lanes 2, 9); **R_2CNA_** (lanes 3, 8); **R_2Peg_** (lanes 4, 7); **R** (lanes 5, 6). The length of the mains fragments is indicated. Inset: gel overexposed.

We investigated CNA within reverse primers as a potential terminator able to block the elongation of the sense strand in a PCR reaction. Two different DNA polymerases, either possessing (*Pfu* DNA polymerase) or lacking (*Taq* DNA polymerase) proofreading activity were used in PCR. Each polymerase was tested in the same PCR conditions on an arbitrary 78 bp template (Fig. 2A) with different reverse primers. In this experiment, the forward primer was ^32^P labeled at the 5′ extremity to detect only the sense strand and PCR products were analyzed using denaturing acrylamide gel electrophoresis. Three PCR controls were performed with three different reverse primers: a short reverse primer included in the template (primer R), a longer reverse primer without modification (primer R_W_), and the primer R_2peg_ containing a terminator composed of two successive triethylene glycol phosphate units. PCR amplification with primer R gave a main signal of 79 nt with the *Taq* DNA polymerase (Fig. 2B, lane 5) and 78 nt with *Pfu* DNA polymerase (Fig. 2B, lane 6). As expected, *Pfu* DNA polymerase synthesised a product corresponding exactly to the length of the template whereas *Taq* DNA polymerase added an extra nucleotide to the 3′ end with its terminal transferase activity (*i.e.* the ability to add an extra base –usually an adenine– at the 3′ ends independently of the template). Some additional bands were visible with a very low intensity and correspond to n−1 and n+1 fragments (see discussion). When the R_W_ primer was used, the addition of an extra nucleotide to the 3′ end of the PCR product was also observed with the *Taq* DNA polymerase (Fig. 2B, lane 1) but not with the *Pfu* DNA polymerase (Fig. 2B, lane 10). These different behaviours of the polymerases have been widely reported in the literature [29], [30], [31]. The third PCR control experiment was performed with the primer R_2peg_ (Fig. 2B, lanes 4 and 7). As expected, both DNA polymerases were never able to bypass this terminator. The results obtained with these three control primers clearly confirmed that the “polishing” activity of *Pfu* DNA polymerase was efficient on blunt and 3′ recessed ends. In contrast, an extra-nucleotide was systematically added with *Taq* DNA polymerase, as a 79 nt fragment was mainly obtained.

To test the polymerisation activity of each DNA polymerase in the presence of CNA, we incorporated one CNA into a central position of the reverse primer (Primer R_CNA_). A single PCR product was observed with the two enzymes (Fig. 2B, lanes 2 and 9). In both cases the polymerases were able to bypass the modification introduced into the R_CNA_ reverse primer. The *Pfu* DNA polymerase synthesized a 96 nt fragment, suggesting that the enzyme was able to skip the CNA otherwise the fragment would have been 98 nt long (Fig. 2A). With the *Taq* DNA polymerase, the amplicons had a length of 97 nt. This suggests that *Taq* DNA polymerase was also able to skip this modification and the supplementary nucleotide was only due to the terminal transferase activity of this enzyme.

**Figure 3:**
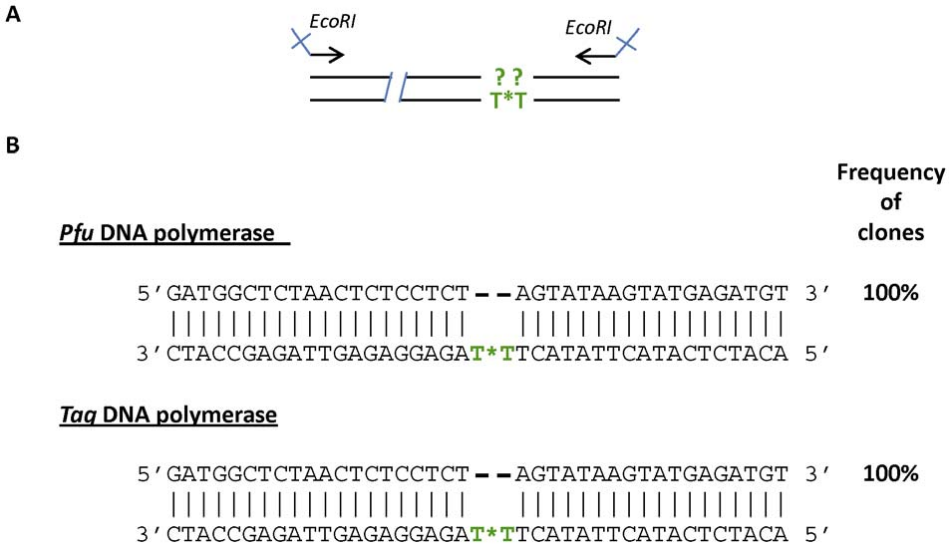
Sequences of sense single-strand DNA obtained with R_CNA_. A) Schematic representation of the PCR realized for cloning. The same DNA polymerase was used in this second PCR than in the first PCR. EcoRI sites incorporated for cloning were indicated. B) Sequences obtained for each polymerase with the R_CNA_ primer.

To precisely analyse the fragments generated by each polymerase, we cloned and sequenced the sense single-strand DNA. PCR products were loaded onto a denaturing acrylamide gel and extracted (Figure S1). We thus set up a cloning strategy to precisely determine which nucleotides were skipped by polymerases in front of the CNA (Fig. 3A). A second PCR was thus performed with primers carrying restriction sites and surrounding the position of the CNA. The reverse primer was chosen so as not to be just after the position of the CNA and the PCR was performed with the same DNA polymerase as the one used during the first PCR. The sequencing (Fig. 3B) confirmed that both enzymes were able to skip the modification introduced into the antisense strand without adding any nucleotide opposite to the CNA. Thus, the structural constraint of the template strand did not block the enzymes, and this single CNA was constantly bypassed.

### Two successive α,β-D-CNA TT units display strong terminator activity

The structural constraint introduced by a single CNA was not sufficient to block polymerases, and we therefore introduced two successive CNA units spaced by one phosphodiester bond into the reverse primer (Primer R_2CNA_, Fig. 2A). Using this reverse primer, the two enzymes displayed completely different behaviours. *Pfu* DNA polymerase was never able to read-through this modification and a unique main signal was detected (Fig. 2B, lane 8). This PCR product was 79 nt long indicating that *Pfu* DNA polymerase incorporated a supplementary nucleotide compared with the product length obtained with the R_2peg_ primer. This addition could be due to the incorporation of a nucleotide in front of the thymidine of the CNA or to the terminal transferase activity of this enzyme which was not compensated by its proofreading activity on this kind of template. However, the insertion of two successive CNA generates a structural constraint which completely stopped the *Pfu* DNA polymerase, as we observed with the two triethylene glycol phosphate units as terminator.

*Taq* DNA polymerase synthesized elongated fragments of different lengths (Fig. 2B, lane 3). With a longer exposure of the gel, a signal can be detected near 98 nt (Fig. 2B, inset). This weak signal indicated that *Taq* DNA polymerase could occasionally synthesize a full-length fragment and consequently that the structural constraint introduced by the two CNA was not sufficient to completely stop this enzyme. Thus the two CNA units inserted into the R_2CNA_ reverse primer displayed dual effects on the behaviour of *Taq* DNA polymerase; either the enzyme passed through the modification or the two CNA terminated polymerization. When this structural constraint was perceived as a terminator by the enzyme, the end point of polymerization spreads out over several nucleotides (Fig. 2B).

**Figure 4:**
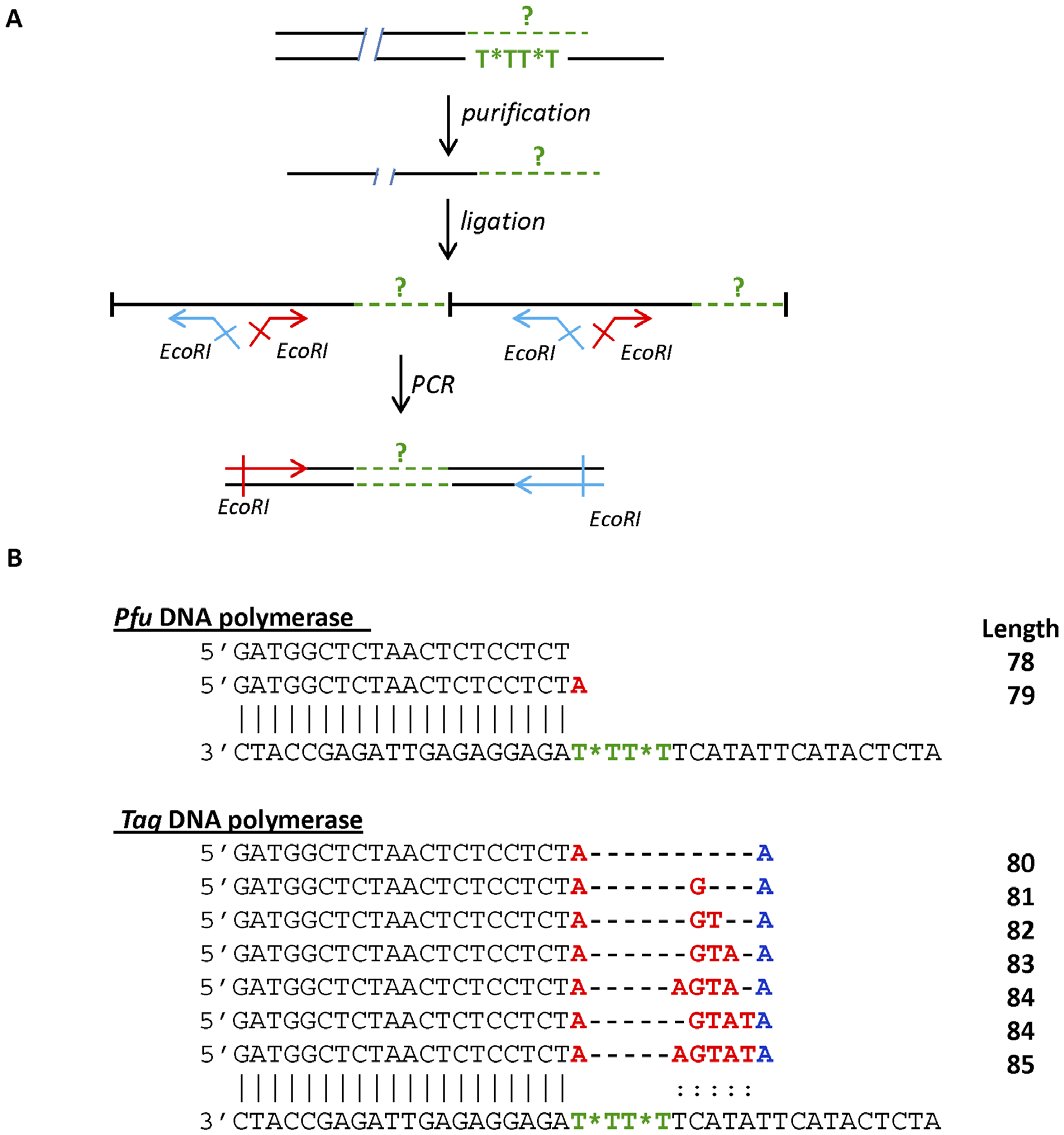
Sequences of sense single-strand DNA obtained with R_2CNA_. A) Schematic representation of the strategy used for cloning. The sense single strand DNA were purified and ligated with RNA ligase. A second PCR was performed with forward (red) and reverse (blue) primers surrounding the position of ligation. These PCR products were then cloned and sequenced. B) Sequences obtained for each polymerase with the R_2CNA_ primer. The nucleotides corresponding to the terminal transferase activity of *Taq* DNA polymerase are indicated in blue and incorporation of nucleotides is shown in red. The length of these fragments is indicated.

The contrasting behaviour between *Taq* DNA polymerase and *Pfu* DNA polymerase with the R_2CNA_ primer was confirmed by sequencing the single-strand DNA. The sense single strands of DNA were purified on a denaturing acrylamide gel (Figure S1). The strategy used for cloning was to ligate the sense single-strand DNA, and thereby generate concatemers independently from the stop point of the enzyme. These concatemers were then amplified by PCR with primers surrounding the position of ligation (Fig. 4A) and these PCR products were cloned and sequenced (Fig. 4B). With *Pfu* DNA polymerase only two kinds of sequence were found. As shown in Figure 2B, the main product is 79 nt long, and this length increase corresponded to the addition of one adenosine to the 3′ end of these fragments. However, a few clones did not present this supplementary nucleotide. *Taq* DNA polymerase partially skipped the modification since one adenosine was systematically incorporated in front of the two CNA units (Fig. 4B). A few nucleotides were then usually incorporated, and in all cases an extra nucleotide was added to the 3′ end of the fragment due to the strong terminal transferase activity of *Taq* DNA polymerase. The maximum length of fragment obtained by cloning was 85 nt and the predominant fragments were between 80 and 82 nt (Fig. 2B). Each single-strand DNA product was cloned three times but the proportion of the various sequences obtained varied greatly from one cloning to the other. We therefore did not extract any statistics from these results and only present the different sequences obtained (see discussion).

## Discussion

We investigated the conformational constraints within single-stranded DNA as a template for DNA polymerases. For this purpose, we used CNA to induce bending in the template. Since they were first synthesized [22], no published work has reported the functional impact of CNA on the behaviour of DNA polymerases. We used two different thermostable DNA polymerases in PCR: *Pfu* DNA polymerase and *Taq* DNA polymerase. The former is a classical enzyme used in PCR for its proofreading activity. It has the lowest error rate of all the commonly used thermostable polymerases [30] and its high-fidelity amplification capacity is provided by its 3′→5′ exonuclease activity (also known as polishing activity [29]). The *Taq* DNA polymerase catalyzes highly accurate DNA synthesis *in vitro*, despite lacking a 3′→5′ proofreading exonuclease activity [31].

To check for the potential terminator function of CNA we performed PCR using a forward primer labelled with ^32^P on its 5′ end to detect only the sense strand with a high sensitivity. With this method, some additional bands were visible with a very low intensity in all conditions tested (Fig. 2B) and correspond to n−1 and n+1 fragments. These n+1 fragments were obtained during the chemical synthesis of long fragments, especially when guanines were incorporated and this modification of length corresponded to a GG dimer formation. Over the large number of couplings in the synthesis of long oligonucleotides, this can lead to a significant n+1 peak. As with the n−1 compounds, these n+1 fragments were difficult to separate from the full-length oligonucleotides (http://www.glenresearch.com//GlenReports/GR21-211.html).

Regardless of proofreading activity, introduction of a single CNA unit within the template induced a conformational stress that was sufficient for both polymerases to bypass the two thymidines. CNA can form loop-like structures [27], [32] and it is clear that the DNA polymerases systematically skip this loop. The mechanism involved in this skipping is unlikely to be a slippage since the template sequence used in our study does not correspond to any of the previously published slippage conditions. In fact, replication slippage [33], [34] is a mechanism that can occur in frameshift mutations of homopolymeric sequences [35] and in deletions between short or long directly repeated sequences [36], [37]. It has been previously shown that thermostable DNA polymerases induce deletions on hairpin structures by a process termed “polymerization across” [38]. We suggest that the same mechanism is involved with the loop-like structure generated with CNA.

When two CNA units are introduced into the reverse primer, the behaviour of the two classes of polymerases differs. The *Pfu* DNA polymerase is completely blocked by this modification, as it does with two triethylene glycol phosphate units. The only difference between these two terminators is that one adenosine is incorporated in front of the CNA. The best yields in chemical synthesis of these CNA are obtained with thymidine as a nucleobase. It was thus very difficult to test nucleotides other than thymidine in these CNA. However, the results obtained with *Taq* DNA polymerase convincingly demonstrate that the incorporation of the additional adenosine by *Pfu* DNA polymerase is due to the presence of one T in the CNA rather than the terminal transferase activity of this enzyme. The sequencing results show that this addition is not systematic and that a small proportion of PCR products were stopped without any supplementary 3′end addition. We did not try to quantitatively express the proportions for each kind of PCR product. Indeed, these clonings were performed several times and a bias was observed since the fraction cloned with adenosine does not reflect the percentage synthesized and observed on the acrylamide gel. We therefore have not presented any statistical data deduced from these clonings and simply report the nature of the sequences obtained. These clonings were performed with a concatemerization step which consists of a ligation between the single strands. Our analyses of this bias lead us to propose two possible hypotheses: the efficiency of this ligation either depends on the 3′ donor, or, for a reason that we are unable to identify, the cloned fraction does not reflect the actual percentage synthesized. These two CNA units thus demonstrate a novel strong terminator activity when a proofreading enzyme such as *Pfu* DNA polymerase is used in PCR.

In contrast, with a non-proofreading enzyme such as *Taq* DNA polymerase, CNA in tandem does not behave as a strong terminator in PCR; *Taq* DNA polymerase never stops precisely at the site of modification, but a few nucleotides later, or more rarely at the end of the template. *Taq* DNA polymerase maintained its activity beyond this modification, whereas *Pfu* DNA polymerase did not. We suggest that this difference of behaviour may be due to the “tightness” and rigidity of the active site of these two polymerases [39]. It follows that, CNA and their derivatives are potentially useful analytical tools to study DNA polymerase behaviour. It would be particularly interesting to study the effects of varying the distance between the two CNA. Finally, similar to abasic triethylene glycol phosphate units, a tandem CNA is a strong terminator in PCR when using a proofreading enzyme such as *Pfu* DNA polymerase. Our results provide the first example of a sugar/phosphate backbone constraint that impacts on enzymatic activity without modifying the nucleobase nature or the sugar structure on single-stranded DNA.

## Materials and Methods

All oligonucleotides were purchased from Sigma-Genosys (St. Quentin Fallavier, France), except for primers containing, α,β-D-CNA and PEG (DMT triethyloxy glycol amidite, GeneCust, Dudelange, Luxembourg), which were synthesized by J-M E. [γ-^32^P]-ATP (6000 Ci/mmol) was purchased from Perkin Elmer (Courtaboeuf, France). *Pfu* DNA polymerase was purchased from Promega (Madison, USA). Recombinant *Taq* DNA polymerase was produced and purified by LP. T4 RNA ligase was purchased from Fermentas (supplied by Euromedex, Mundolsheim, France). All other enzymes were purchased from New England Biolabs (Ozyme, St-Quentin-en-Yvelines, France). Cogenics (Meylan, France) performed DNA sequencing.

### Synthesis of can

The synthesis of (*S*_C5′_, R*_P_*) α,β-D-Constrained Nucleic Acids has been previously reported [22]. In the primer sequences, α,β-D-CNA TT is indicated as T*T and triethylene glycol phosphate unit is indicated as Peg. Incorporation of (*S*_C5′_, R*_P_*) α,β-D-CNA TT was verified by MALDI-TOF MS (Data not shown).

### Polymerase Chain Reaction

The PCR reaction mixtures were prepared in a total volume of 50 µL. For each DNA polymerase (1.5units) , the same conditions were used: 250 µM of each dNTP, 0.5 µM of reverse and forward primers (Table 1), 40 nM of DNA template (T: 5′-GGTATTGAGGGTCGCATCTGGGGGGGATGTATTCTGGGGGGTTGGGCCGGGGTCCCCGGATGGCTCTAACTCTCCTCT-3′, 78mer) in the appropriate reaction buffer [*10× Pfu buffer*: 200 mM Tris-HCl (pH 8.8 at 25°C), 100 mMKCl, 100 mM (NH_4_)_2_SO_4_, 20 mM MgSO_4_, 1% TritonX-100 and 1 mg/mL nuclease-free BSA. *10× Taq buffer*: 100 mM Tris-HCl (pH 8,3 at 25°C), 500 mM KCl, 15 mM MgCl_2_, 0.1% gelatine.]. Forward primer was end-labelled by T4 polynucleotide kinase with ATP-γ ^32^P, and then purified by size exclusion using Microspin G-25 columns (GE Healthcare). Amplifications were performed on GeneAmp® PCR system 9700 from Applied Biosystems, with the following parameters: 94°C 2 min (94°C 30 s; 60°C 20 s; 72°C 10 s) for 30 cycles, followed by an extension step at 72°C for 1 min. PCR products were loaded onto denaturing urea-polyacrylamide gel and migration was performed at constant power (20 W). The gel was dried and exposed using Kodak Biomax film overnight at room temperature.

**Table 1:** Sequences of the primers used in PCR.

Primer	Sequence
**F**	5′-ggtattgagggtcgcatc-3′
**R**	5′-agaggagagttagagccatc-3′
**R_W_**	5′-acatcgcatacttatact **T** agaggagagttagagccatc-3′
**R_CNA_**	5′-acatctcatacttatact T*T agaggagagttagagccatc-3′
**R_2CNA_**	5′-atctcatacttatact T*TT*T agaggagagttagagccatc-3′
**R_2Peg_**	5′-atctcatacttatact **PegPeg** agaggagagttagagccatc-3′

α,β-D-CNA TT is indicated by T*T and triethylene glycol phosphate unit by **Peg** in the primer sequences. The modification of lengths due to the insertion of α,β-D-CNA TT or triethylene glycol phosphate unit in primers **R_CNA_** and **R_2CNA_**, correspond to the addition of 2, and 4 nucleotides equivalent, respectively.

### Purification of (+) strand ssDNA

PCR products were loaded onto denaturing urea-polyacrylamide gel and strands of unequal length were separated by electrophoresis and purified. Samples were prepared in 2× denaturing loading buffer (20 mM EDTA, 0.05% Xylene cyanol, prepared in 95% formamide), heated for 5 minutes at 95°C and transferred onto ice to prevent annealing. Electrophoresis was carried out in a 10% polyacrylamide (19∶1)-7M urea gel prepared in TBE 1× (100 mM Tris pH 8.4, 90 mM Boric acid and 1 mM EDTA). A pre-run of 30 minutes at constant power (25 W) was performed before denatured samples were loaded and electrophoresed at constant power (20 W) for ≈40 minutes. Gels were stained with ethidium bromide (0.5 µg/ml) and DNA was visualized under UV light.

To purify the band corresponding to the strand (+), DNA was excised and eluted for 2 h 65°C in 300 µl of TAE 1× and purified using DNA gel extraction kit (Millipore) and quantified at 260 nm.

### Ligation of (+) strand ssDNA

(+) strand ssDNA, obtained with R_2CNA_ and R_2Peg_ reverse primers, was purified and 5′-phosphorylated using T4 polynucleotide kinase. The reaction mixtures were prepared in a final volume of 10 µL with 4 µL of gel-extracted ssDNA (20–40 ng), ATP 1 mM, BSA 0.1 mg.mL^−1^, 10 U T4 PNK in T4 RNA ligase buffer 1×, then incubated at 37°C for 1 h. The 5′-P ssDNA fragments were concatemerized at 16°C overnight in a total volume of 11 µL by the addition of 1 U T4 RNA ligase.

### PCR generation of EcoRI-ended fragments from purified ssDNA amplified with R_2Peg_ or R_2CNA_ reverse primers

PCR using EcoRI forward and reverse primers (Table 2) were performed in a total reaction volume of 50 µL with *Taq* Pol buffer or *Pfu* buffer containing each primer (0.5 µM), each dNTP (250 µM), 4 µL of ssDNA concatemers and 1.5 U of the corresponding polymerase. 30 PCR cycles of 30 s at 94°C, 20 s at 60°C and 10 s at 72°C were performed. A final extension was then carried out for 1 min at 72°C.

**Table 2:** Sequences of the primers used for cloning.

Primer	Sequence	Length
EcoRI forward primer	5′-ATCGGAATTCGGGATGTATTCTGGG-3′	25mer
EcoRI reverse primer	5′-TACGGAATTCCCCCAGATGCGACCC-3′	25mer
EcoRI _CNA_ forward primer	5′-atcgatcatcgaattcggtattgagggtcgcatc-3′	34mer
EcoRI _CNA_ reverse primer	5′-TACGGAATTCACATCTCATACTTA-3′	24mer

EcoRI digestion sites are underlined.

### PCR generation of Eco RI ended fragments amplified with R_CNA_ reverse primer

PCR products obtained with R_CNA_ reverse primer were amplified by a second PCR using EcoRI_CNA_ forward and reverse primers (Table 2), without ssDNA purification. The second PCR was performed with the polymerase used in the first PCR.

### Cloning

EcoRI-ended fragments were digested by EcoRI in buffer (50 mM NaCl, 100 mM Tris-HCl, 10 mM MgCl2, 0,025% triton X100) for 1 h at 37°C, precipitated and ligated by T4 DNA ligase into pBluescript SK^−^ vector (Stratagene) EcoRI digested and CIP (Ozyme) dephosphorylated. The ligation reaction was transformed into XL1blue *E. coli* strain. Transformed bacteria were plated on Mac Conkey medium supplemented with ampicillin (100 µg/ml) and incubated overnight at 37°C.

## Supporting Information

Figure S1 **Purification of single strands DNA for cloning.** PCR products were loaded onto two low resolution denaturing acrylamide gels. After electrophoresis, one gel was transferred on Nylon membrane and hybridized with an antisense probe to localize the sense single strands of DNA. The pieces of the second gel corresponding to the hybridization area were cut and the DNA extracted for cloning. (A) Denaturing Polyacrylamide Gel Electrophoresis obtained for each set of primers with *Pfu* DNA polymerase and *Taq* DNA polymerase, stained with ethidium bromide. For each polymerase, the PCR were realized with different reverse primers: **R_W_** (lanes 1, 10); **R_CNA_** (lanes 2, 9); **R_2CNA_** (lanes 3, 8); **R_2Peg_** (lanes 4, 7); **R** (lane 6). Lane 5 (**T**) corresponds to the 78 bp template. The length of the mains fragments is indicated. (B) Hybridization with antisense probe of gel shown in (A). Denaturing urea-polyacrylamide gels were transferred by capillarity on Nylon membrane in 20× SSC over night at room temperature, then UV crosslinked using Spectrolinker (XL1000). The membrane was prehybridized 1 h at 60°C in Church buffer (0.5 M phosphate buffer pH 7.5, 7% SDS, 1% BSA, 1 mM EDTA). Hybridization was performed in the same buffer at 60°C overnight with 2 ng of labelled antisense probes (5′-TGGGGGGGATGTATTCTGGGGGGTTGGGCCGGGGTCCCCG-3′, 40mer). Probes were end-labelled by T4 polynucleotide kinase with ATP γ^32^P, then purified by size exclusion using Microspin G-25 columns (GE Healthcare). Specific activity was evaluated at about 5 10^8^ cpm/µg. After hybridization, the membrane was washed twice with SSC 0.2×, SDS 0.1%, 15 min at 60°C, and exposed using Kodak film Biomax over night at room temperature.
(TIF)
