# Supplementary figures and images for: α,β-D-Constrained Nucleic Acids Are Strong Terminators of Thermostable DNA Polymerases in Polymerase Chain Reaction
Source: PLoS One. 2011 Oct 3;6(10):e25510. doi: 10.1371/journal.pone.0025510 (PMC3185000; doi:10.1371/journal.pone.0025510)

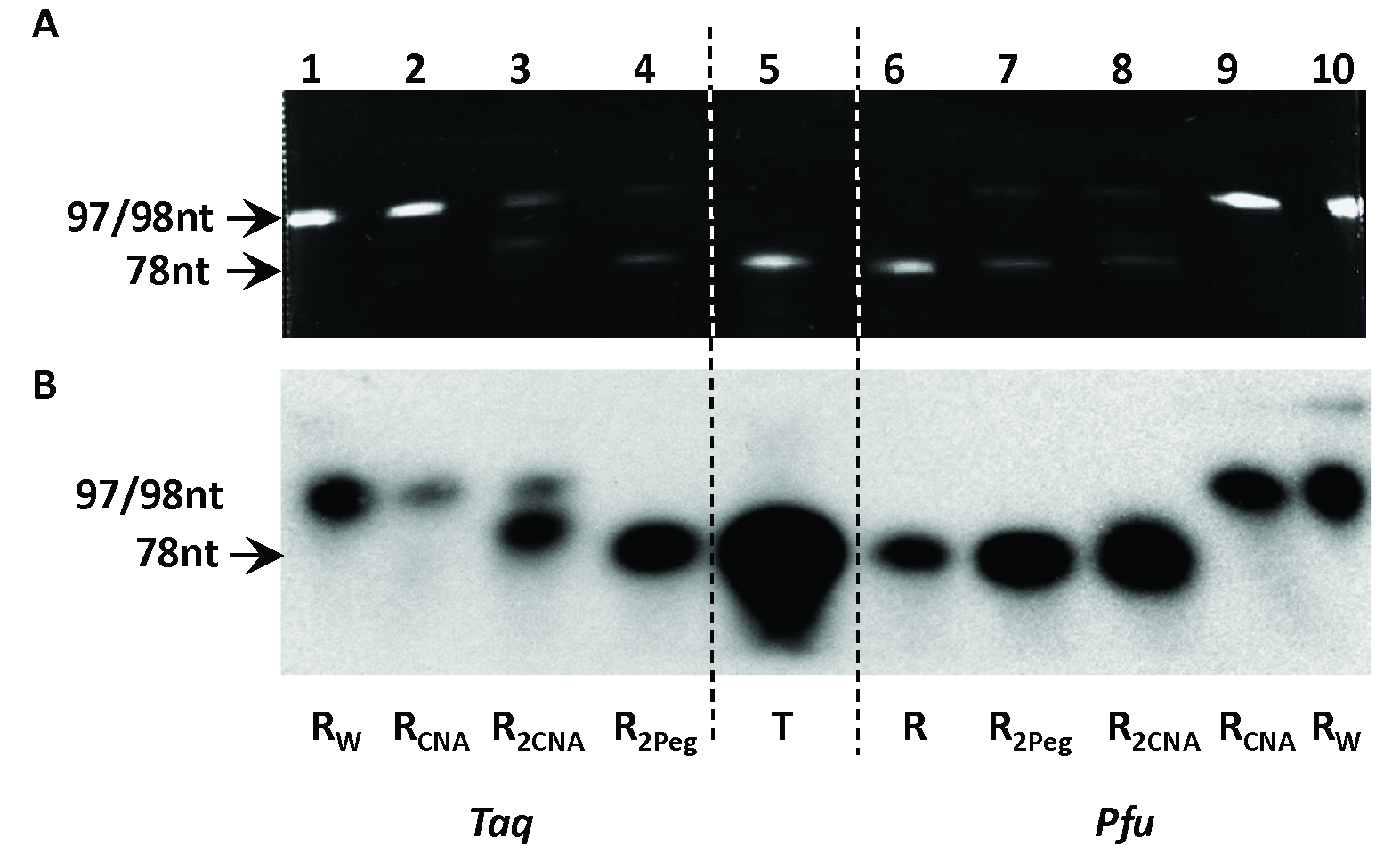

Supplement: Figure S1 — Purification of single strands DNA for cloning. PCR products were loaded onto two low resolution denaturing acrylamide gels. After electrophoresis, one gel was transferred on Nylon membrane and hybridized with an antisense probe to localize the sense single strands of DNA. The pieces of the second gel corresponding to the hybridization area were cut and the DNA extracted for cloning. (A) Denaturing Polyacrylamide Gel Electrophoresis obtained for each set of primers with Pfu DNA polymerase and Taq DNA polymerase, stained with ethidium bromide. For each polymerase, the PCR were realized with different reverse primers: RW (lanes 1, 10); RCNA (lanes 2, 9); R2CNA (lanes 3, 8); R2Peg (lanes 4, 7); R (lane 6). Lane 5 (T) corresponds to the 78 bp template. The length of the mains fragments is indicated. (B) Hybridization with antisense probe of gel shown in (A). Denaturing urea-polyacrylamide gels were transferred by capillarity on Nylon membrane in 20× SSC over night at room temperature, then UV crosslinked using Spectrolinker (XL1000). The membrane was prehybridized 1 h at 60°C in Church buffer (0.5 M phosphate buffer pH 7.5, 7% SDS, 1% BSA, 1 mM EDTA). Hybridization was performed in the same buffer at 60°C overnight with 2 ng of labelled antisense probes (5′-TGGGGGGGATGTATTCTGGGGGGTTGGGCCGGGGTCCCCG-3′, 40mer). Probes were end-labelled by T4 polynucleotide kinase with ATP γ32P, then purified by size exclusion using Microspin G-25 columns (GE Healthcare). Specific activity was evaluated at about 5 108 cpm/µg. After hybridization, the membrane was washed twice with SSC 0.2×, SDS 0.1%, 15 min at 60°C, and exposed using Kodak film Biomax over night at room temperature. (TIF) [file pone.0025510.s001.tif]
